# Discovery of CLEC2B as a diagnostic biomarker and screening of celastrol as a candidate drug for psoriatic arthritis through bioinformatics analysis

**DOI:** 10.1186/s13018-023-03843-0

**Published:** 2023-05-29

**Authors:** Min Niu, Jingman Yuan, Meixi Yan, Ge Yang, Ziyi Yan, Xichao Yang

**Affiliations:** Department of Rheumatology Immunology and Endocrinology, Honghui Hospital, Xi’an Jiaotong University, Xi’an, 710061 Shaanxi China

**Keywords:** Psoriatic arthritis, Celastrol, Bioinformatics analysis, Molecular docking, Psoriasis

## Abstract

**Background:**

Psoriatic arthritis (PSA) is a chronic, immune-mediated inflammatory joint disease that is liked to mortality due to cardiovascular disease. Diagnostic markers and effective therapeutic options for PSA remain limited due to the lack of understanding of the pathogenesis. We aimed to identify potential diagnostic markers and screen the therapeutic compounds for PSA based on bioinformatics analysis.

**Methods:**

Differentially expressed genes (DEGs) of PSA were identified from the GSE61281 dataset. WGCNA was used to identify PSA-related modules and prognostic biomarkers. Clinical samples were collected to validate the expression of the diagnostic gene. These DEGs were subjected to the CMap database for the identification of therapeutic candidates for PSA. Potential pathways and targets for drug candidates to treat PSA were predicted using Network Pharmacology. Molecular docking techniques were used to validate key targets.

**Results:**

CLEC2B was identified as a diagnostic marker for PSA patients (AUC > 0.8) and was significantly upregulated in blood samples. In addition, celastrol was identified as a candidate drug for PSA. Subsequently, the network pharmacology approach identified four core targets (IL6, TNF, GAPDH, and AKT1) of celastrol and revealed that celastrol could treat PSA by modulating inflammatory-related pathways. Finally, molecular docking demonstrated stable binding of celastrol to four core targets in the treatment of PSA. Animal experiments indicated celastrol alleviated inflammatory response in the mannan-induced PSA.

**Conclusion:**

CLEC2B was a diagnostic marker for PSA patients. Celastrol was identified as a potential therapeutic drug for PSA via regulating immunity and inflammation.

**Supplementary Information:**

The online version contains supplementary material available at 10.1186/s13018-023-03843-0.

## Introduction

Psoriatic arthritis (PSA) is an immune-related inflammatory musculoskeletal disorder with unclear etiology. It impacts 2–3% of the population [1]. PSA is a multifaceted disease that affects several organ systems. It is associated with co-morbidities such as cardiovascular disease, inflammatory bowel disease, uveitis, and osteoporosis [2]. This heterogeneity makes PSA difficult to diagnose clinically. At present, there are no biomarkers for the diagnosis of PSA patients. Therefore, in-depth research into the pathogenesis of PSA can contribute to the development of novel diagnostic markers that will facilitate the development of effective biologics and small-molecule drugs to prevent disease progression and improve quality of life [3].

Although the pathogenesis of PSA is not completely understood, researchers revealed that PSA is intimately linked with immune system dysfunction and inflammation [4, 5]. In patients with PSA, the systemic immune inflammation index may provide a simple, convenient, and cost-effective means of monitoring disease activity and treatment response [6]. A previous study identified IL-17A as the key effector cytokine, which drives PSA pathogenesis [7]. In recent years, the most interesting targets in PSA have been the IL-17/IL-23 pathway and Th-17 cells [8]. A novel anti-PSA drug such as ustekinumab has been developed as a result [9]. For patients with PSA, IL-36 antagonists may be a promising new therapeutic target [10]. Different clinical manifestations of PSA may benefit from anti-TNF-α therapy [11]. These findings suggested that anti-inflammatories may reduce the release of inflammatory factors, thereby alleviating PSA. Therefore, natural compounds that are effective and safe for the treatment of PSA through the inhibition of inflammation should be investigated.

As a source of medicines, natural herbs are widely used. Due to their good therapeutic properties and multi-target efficacy, these herbs are of interest to scientists [12, 13]. Celastrol has attracted considerable attention in recent years as a plant-derived compound that has exhibited great potential in the treatment of various diseases, including osteoporosis, cholestasis, atherosclerosis, type 2 diabetes, heart failure, obesity, and cerebrovascular injury [14, 15]. Celastrol has been shown to improve psoriasiform inflammation by targeting the IRF1/GSTM3 axis [16]. Celastrol gel decreased the generation of IL-23 from Langerhans cells to improve psoriasis [17]. In addition, celastrol could alleviate rheumatoid arthritis through the activation of autophagy by suppressing the PI3K/AKT pathway [18]. Celastrol inhibited the NF-кB pathway and suppressed the activation of the NLRP3 inflammasome in rheumatoid arthritis [19]. However, the role of celastrol in the treatment of PSA remains unknown.

In the present study, differentially expressed analysis and WGCNA were applied to screen potential diagnostic biomarkers for PSA patients. The CMap database was used to identify celastrol as a therapeutic compound against PSA. Network pharmacological analysis of celastrol’s key targets and pathways and preliminary investigation of the molecular mechanism of celastrol against PSA.

## Materials and methods

### GEO dataset collection

The original gene expression profile was downloaded from the GEO database (GSE61281). The GSE61281 dataset contained 12 control samples and 20 PSA samples. The original matrix data was extracted using the AFFY package. The mRNA probes were annotated using the annotation files and probes were transformed into gene symbols.

### Differential expression analysis between the control and PSA groups

The “limma” package was applied to screen for differentially expressed genes (DEGs) between the control and PSA groups. *p*-value < 0.05 and ∣log fold change∣ ≥ 1 were used as the screening criteria for DEGs identification. The volcano plot was drawn using the “ggplot2” package. Functional enrichment analysis was performed using Metascape to investigate the biological functions and pathways of the DEGs.

### Gene set enrichment analysis (GSEA)

To identify the most significant pathways between the control and PSA groups, the “clusterprofiler” package was used to perform the GSEA. From the Molecular Signatures Database, the subset “h.all.v7.4.symbols.gmt” was downloaded. Statistical significance was defined as *p*-value < 0.05.

### Immune cell infiltration analysis

The xCell was used to evaluate the abundance of immune infiltrating cells in each sample. The results were visualized using “ggpolt2” package.

### Weighed gene co-expression network analysis (WGCNA)

For each gene, we calculated the mean absolute deviation (MAD) based on the gene expression profiling, removing the top 50% of genes with the lowest MAD. The goodSamplesGenes method was then used to remove abnormal genes and samples, and the WGCNA package was applied to construct the co-expression networks. Hub genes were identified as the genes with the highest level of connectivity.

### Quantitative real-time polymerase chain reaction (qRT-PCR)

Blood samples from 10 healthy controls and 10 PSA patients were collected from the Honghui Hospital, Xi’an Jiaotong University. This study was approved by the Ethics Committee of the Honghui Hospital, Xi’an Jiaotong University. Following the manufacturer’s protocols, total RNA was extracted from blood samples using TRIzol reagent (Invitrogen, USA). 2 µg of purified RNA was synthesized into the cDNA using the cDNA synthesis kits (Invitrogen, CA, USA) were used to synthesize RNA into cDNA. A StepOne real-time PCR system (Applied Biosystems, CA, USA) was then used for qRT-PCR. The mRNA expression levels were measured using the 2^–ΔΔCt^ method. Primers were shown in Additional file 1: Table S1.

### Prediction of potential drugs using connectivity map (CMap)

CMap (https://clue.io/data), is a predictive analytical platform used to identify potential drugs for their ability to activate or repress pre-specified genes [20]. A connectivity score of less than − 90 is an indication that the small molecule has potential pharmacological activity [21]. In this study, to identify potential natural compounds that may suppress the biological progression of PSA, we uploaded DEGs from the control and PSA groups into the CMap database.

### PSA-related targets acquisition

A search of the GeneGards (https://www.genecards.org/), Comparative Toxicogenomics Database (https://ctdbase.org/), and Therapeutic Target Database (https://db.idrblab.net/ttd/) was performed using the keyword “psoriatic arthritis”. The targets obtained from the three databases and the DEGs targets were combined and de-duplicated to obtain PSA disease-related targets.

### Celastrol-related targets collection

Celastrol-related targets collected from the HERB database (http://herb.ac.cn/), Super-PRED (https://prediction.charite.de/index.php), SEA Search Server (https://sea.bkslab.org/), and SwissTargetPrediction (http://www.swisstargetprediction.ch/). Then, the targets obtained from those databases were combined and de-duplicated to obtain celastrol-related targets.

### Protein–protein interaction (PPI) network construction

The potential targets of celastrol were mapped to the targets of PSA using a Venn tool to obtain the common targets of celastrol and PSA. Then, we used the STRING database (https://cn.string-db.org/) to build a PPI network for those common targets. The PPI network was imported into the Cytoscape (version 3.7.2) software for visual analysis and the cytoHubba plug-in was used for network critical target screening.

### Functional enrichment analysis of common targets for celastrol and PSA disease

In this study, GO function and KEGG signaling pathway enrichment analysis were performed to further explore the mechanism of celastrol in treating PSA. The ClusterProfiler package was downloaded by Bioconductor, and GO and KEGG enrichment analysis of common targets were performed using R software to set a threshold of *p* < 0.05.

### Molecular docking analysis

The Protein Data Bank (PDB) database (https://www.rcsb.org) was used to download the structure of proteins. The structure of small molecule drugs was downloaded from The PubChem database (https://pubchem.ncbi.nlm.nih.gov/) was applied to download the structure of small molecule compounds. Molecular docking analysis was carried out with AutoDock and the visualization of results with PyMOL software.

### Animal experiments

We purchased mice (C57BL/6 J and 12 weeks of age) from the Experiment Animal Center of Xi’an Jiaotong University. Mice were housed in an air-conditioned room (relative humidity 45–65% and temperature 21–25 °C) with a 12 h dark/light cycle and fed tap water and food ad libitum. The animal experimental protocol was approved by the Ethics Committee of the Honghui Hospital.

Mice were used to induce the mannan-induced psoriatic arthritis (MIP) model according to a widely used protocol [22]. The model of PSA was established through a single intraperitoneal injection of 10 mg of Saccharomyces cerevisiae mannan (Sigma-Aldrich). All mice were divided into five groups (n = 8 per group): normal control group (NC), MIP, MIP group of mice received with celastrol at a dose of 0.5 mg/kg (MIP + LCel), MIP group of mice received with celastrol at a dose of 1 mg/kg (MIP + MCel), MIP group of mice received with celastrol at a dose of 2 mg/kg (MIP + HCel). The dose of celastrol was based on previous study [18]. In order to prepare drugs as suspensions, 0.5% sodium carboxymethyl cellulose (CMC-Na) was used. Celastrol was administered intragastrically once per day from days 1 to 10 post immunization. The NC and MIP group mice, on the other hand, were given 0.5% CMC-Na intragastrically on the same schedule. The standardized macroscopic scoring system was used to blindly assess the mice for both arthritic symptoms and psoriatic lesions [23–25]. On day 10 of the experiment, blood samples were collected and serum was subsequently isolated. Then, the IL-6, IL-1β, and TNF-α level of the serum was measured using enzyme-linked immunosorbent assay (ELISA) based on the manufacturer’s protocols (ThermoFisher, MA, USA).

### Statistical analysis

The data analysis was conducted using GraphPad Prism 8.0 software. The experimental data was reported as mean ± standard deviation (SD). A t-test was utilized to compare data between the two groups. A significance level of *p* < 0.05 was used to indicate statistical significance.

## Results

### Identifying DEGs in PSA

**Fig. 1:**
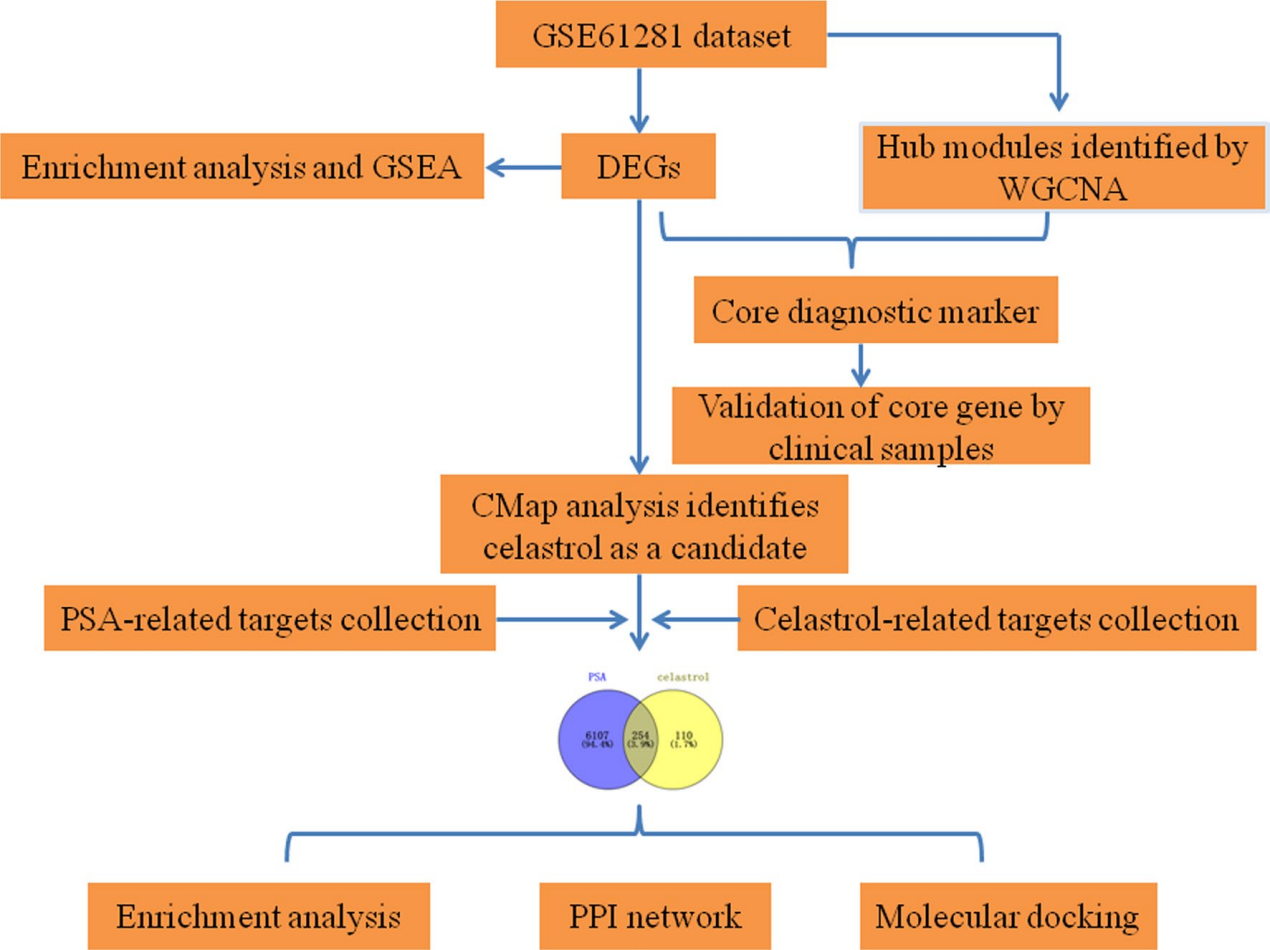
The workflow of this study

**Fig. 2:**
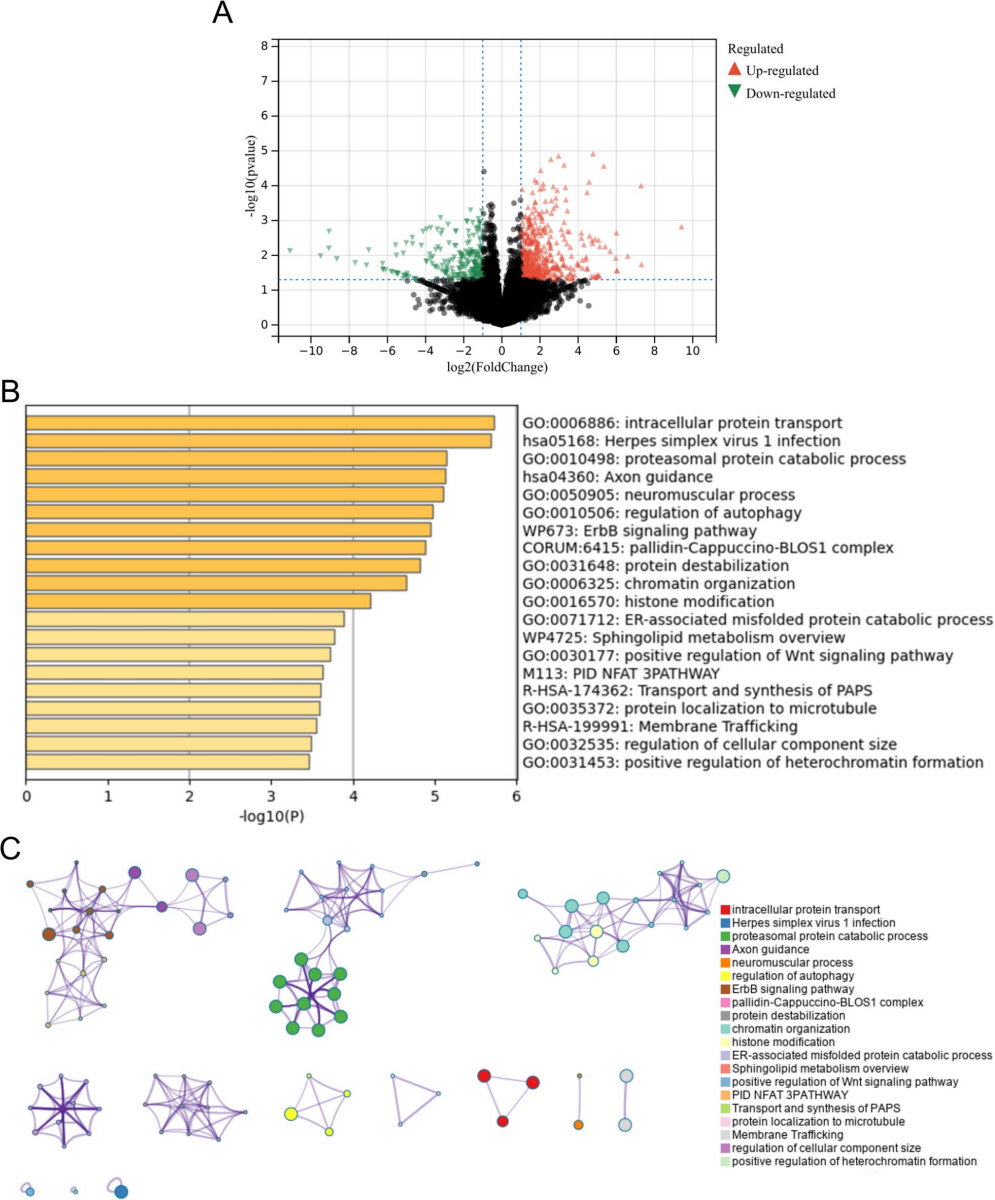
Identification of DEGs in PSA. **A** Volcano presented the DEGs between the control and PSA groups. **B**, **C** Functional enrichment analysis of DEGs using Metascape

The workflow of the present study was presented in Fig. 1. Raw gene expression profiles for the GSE61281 dataset were obtained from the GEO database containing gene microarray data from 20 PSA patients and 12 healthy individuals. A total of 734 DEGs (497 up-regulated genes and 237 down-regulated genes) were identified using the “limma” package (Fig. 2A). In addition, enrichment analysis results indicated those DEGs were significantly enriched in intracellular protein transport, herpes simplex virus 1 infection, proteasomal protein catabolic process, neuromuscular process, regulation of autophagy, ErbB signaling pathway, etc. (Fig. 2B and C).

### GSEA and immune cell infiltration

**Fig. 3:**
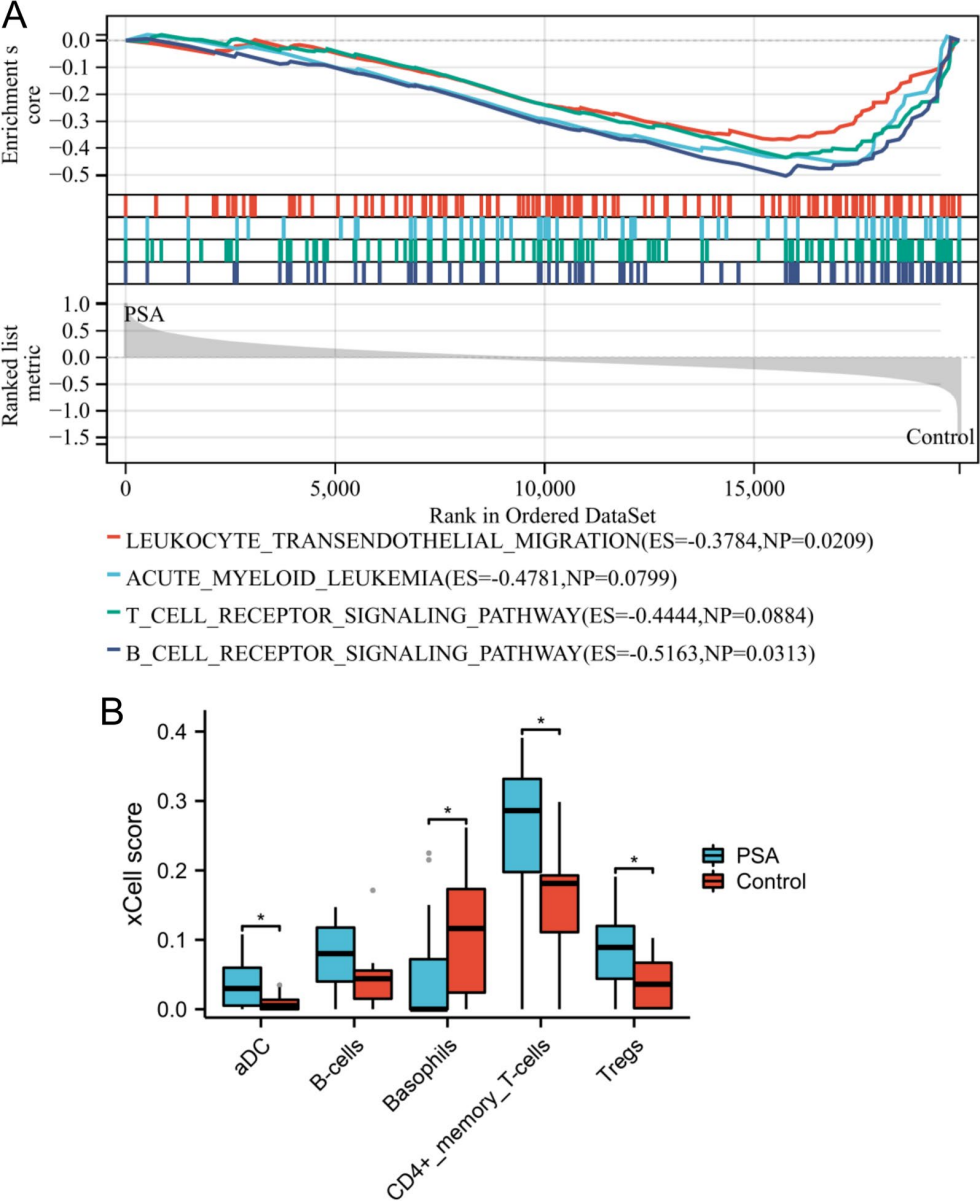
GSEA and immune cell infiltration analysis. **A** The GSEA plot exhibited the significantly enriched gene sets between the control and PSA groups. **B** The boxplot presented the different levels of immune cell infiltration between the control and PSA group

As shown in Fig. 3A, GSEA results revealed that leukocyte transendothelial migration, acute myeloid leukemia, T cell receptor signaling pathway, and B cell receptor signaling pathway were significantly enriched in the control group. In addition, the proportion of basophils was lower in the PSA group than in the control group, whereas the proportion of aDC, CD4 + memory T cells, and Tregs was significantly higher in the PSA group than in the control group (Fig. 3B). These findings suggested that the immune cell process may be implicated in the development of PSA.

### Identification of DEGs in the hub modules

**Fig. 4:**
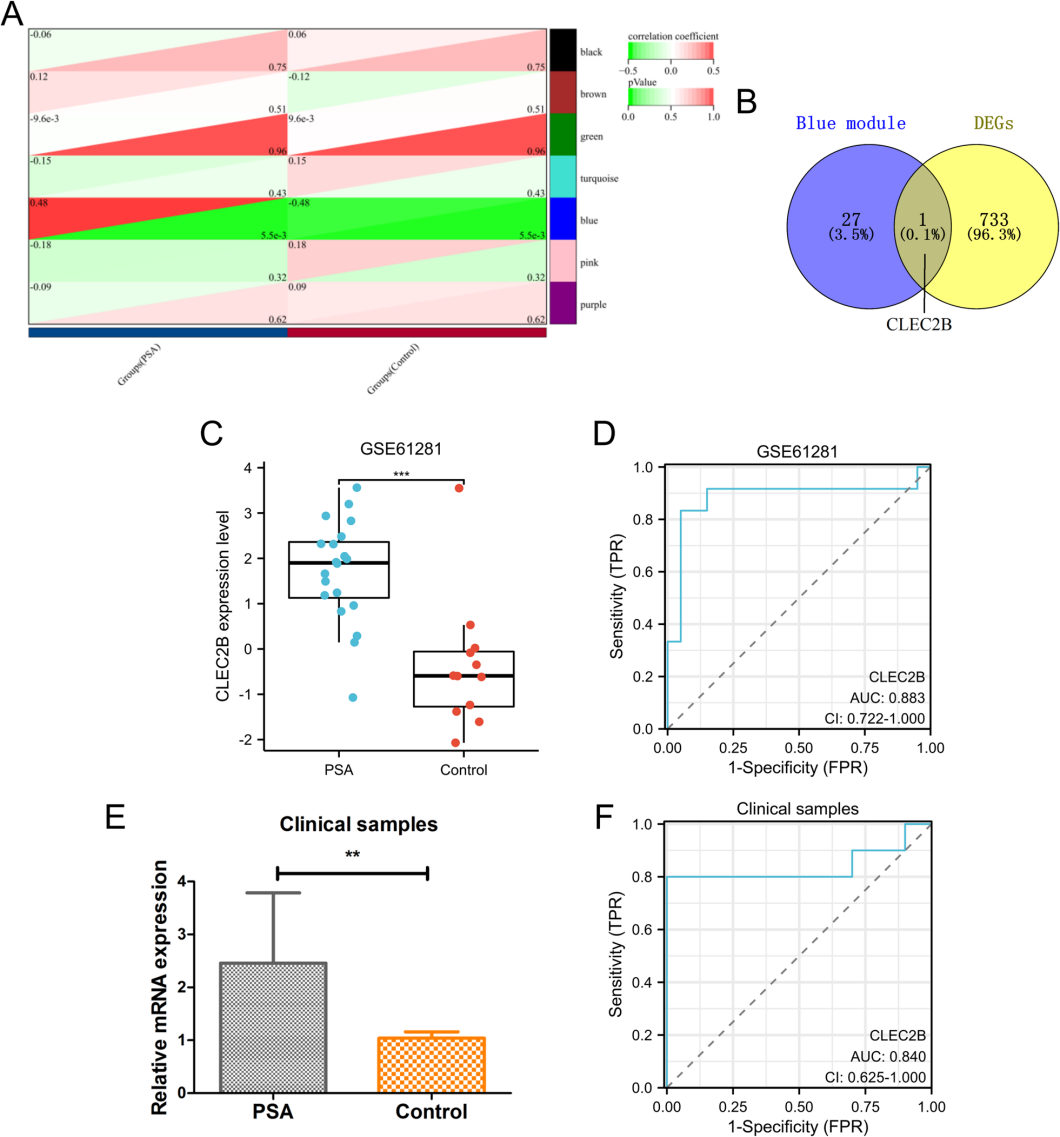
Identification of DEGs in the hub modules. **A** WGCNA identifies the key modules. **B** Intersection gene of DEGs and blue module. The gene expression level **C** and ROC curve **D** of CLEC2B in the GSE61281 dataset. The gene expression level **E** and ROC curve **F** of CLEC2B in clinical samples

As shown in Fig. 4A, 7 gene modules were identified by WGCNA. Among these modules, the blue (r = 0.48, *p* = 5.5e-3) module exhibited a significant correlation with PSA. For further analysis, 28 hub genes were identified in the blue module (Additional file 1: Table S2). As shown in Fig. 4B, CLEC2B was identified from DEGs based on the Venn result. In addition, the expression of CLEC2B was significantly up-regulated in the PSA group in both GSE61281 (*p* < 0.001) and clinical samples (*p* < 0.01) (Fig. 4C and E). Furthermore, the diagnostic AUC value of the CLEC2B gene was 0.88 in GSE61281 (Fig. 4D) and 0.84 in clinical samples (Fig. 4F). These results indicated that CLEC2B gene was a potential biomarker for PSA patients.

### Screening of natural compounds for PSA treatment using CMap

**Fig. 5:**
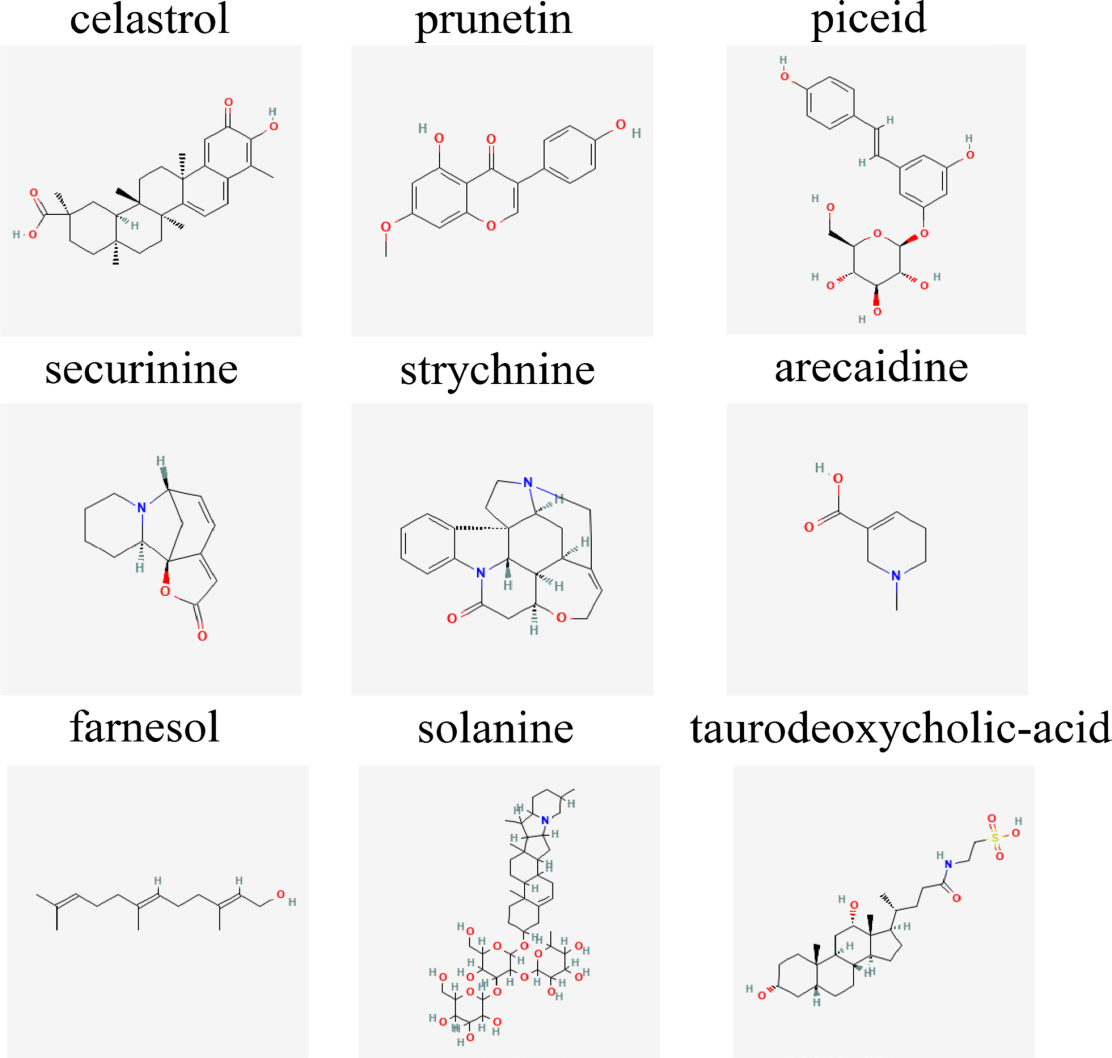
Screening of natural compounds for PSA treatment using CMap

A total of nine natural compounds (celastrol, prunetin, piceid, securinine, strychnine, arecaidine, farnesol, solanine, and taurodeoxycholic-acid) with the highest negative score were evaluated as potential drugs for PSA, as shown in Additional file 1: Table S3. Figure 5 presented the 2D chemical structure of those drug candidates. Among them, celastrol had the lowest score. Furthermore, previous studies indicated celastrol could improve psoriasiform inflammation and rheumatoid arthritis [16, 19]. Therefore, we chose celastrol as a potential anti-PSA agent. We investigated its potential pharmacological mechanisms and targets using network pharmacology and molecular docking analyses.

### Screen potential targets for celastrol in the treatment of PSA

**Fig. 6:**
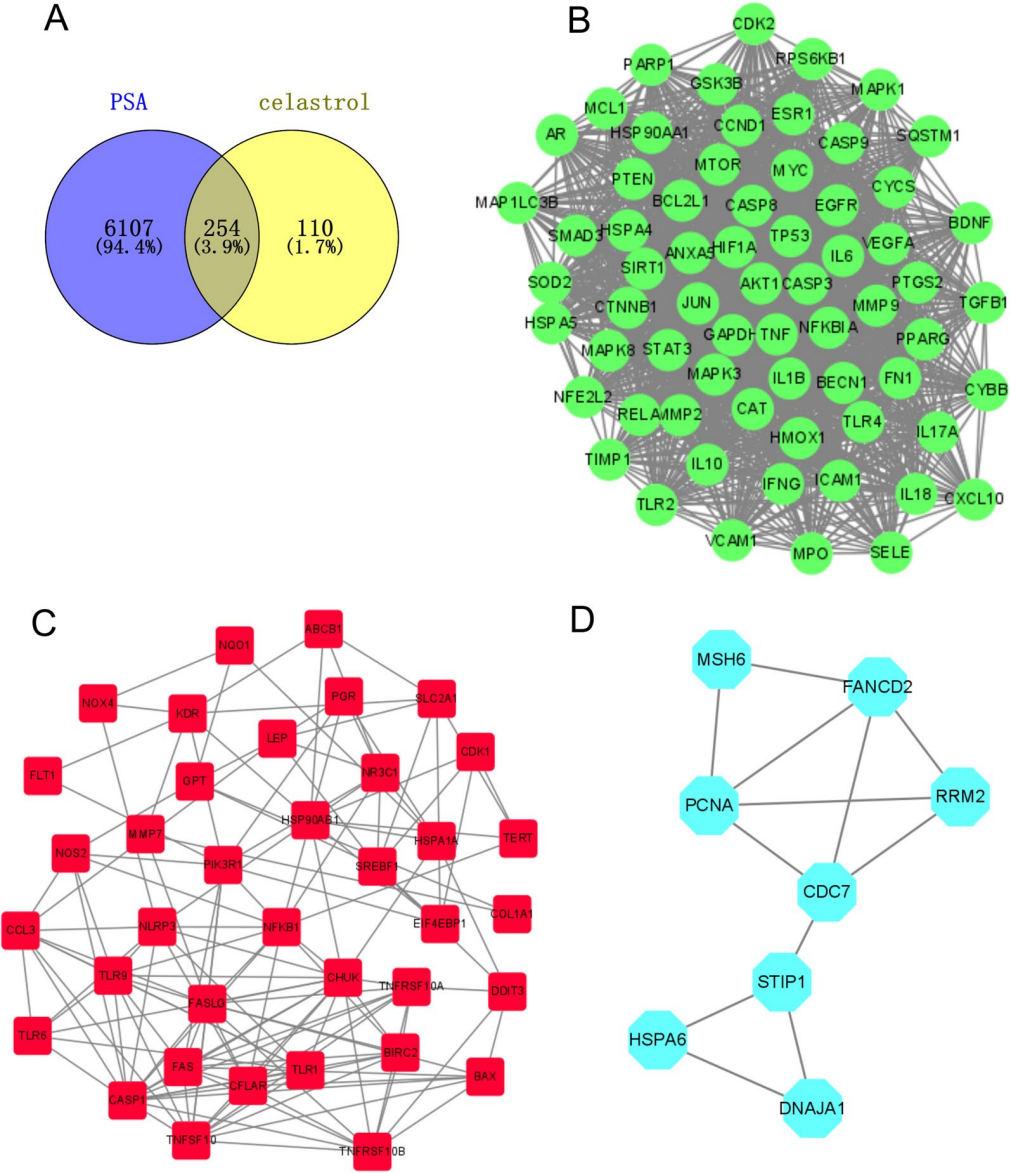
Protein–protein interaction (PPI) network for celastrol against PSA. **A** Intersection genes of PSA-related genes and celastrol-related genes. MCODE plugin was used to construct Cluster 1 (**B**), Cluster 2 (**C**), and Cluster 3 (**D**)

A total of 6361 PSA-related genes were collected from the GeneGards, Comparative Toxicogenomics Database, and Therapeutic Target Database. In addition, we obtained 364 celastrol-related genes from the HERB database, Super-PRED, SEA Search Server, DEGs, and SwissTargetPrediction. Finally, 254 intersection genes were identified as potential targets of celastrol in the treatment of PSA (Fig. 6A).

### PPI network analysis and identification of core sub-networks

We constructed a PPI network using Cytoscape software to visualize the interactions of these 254 overlap genes. In addition, the PPI network was clustered into three clusters using the MCODE algorithm. Cluster 1 contained 66 nodes and 1700 edges (Fig. 6B); Cluster 2 contained 37 nodes and 141 edges (Fig. 6C); Cluster 3 contained 8 nodes and 12 edges (Fig. 6D). Cluster 1 was the primary cluster of the PPI, which may be an important regulatory network for celastrol in PSA treatment. Furthermore, four core targets (IL6, TNF, GAPDH, and AKT1) were identified by stress, radiality, MNC, DMN, MCC, EPC, EcCentricity, degree, closeness, bottleneck, and betweenness algorithms in CytoHubba (Additional file 1: Fig. S1).

### Enrichment analysis of cluster 1

**Fig. 7:**
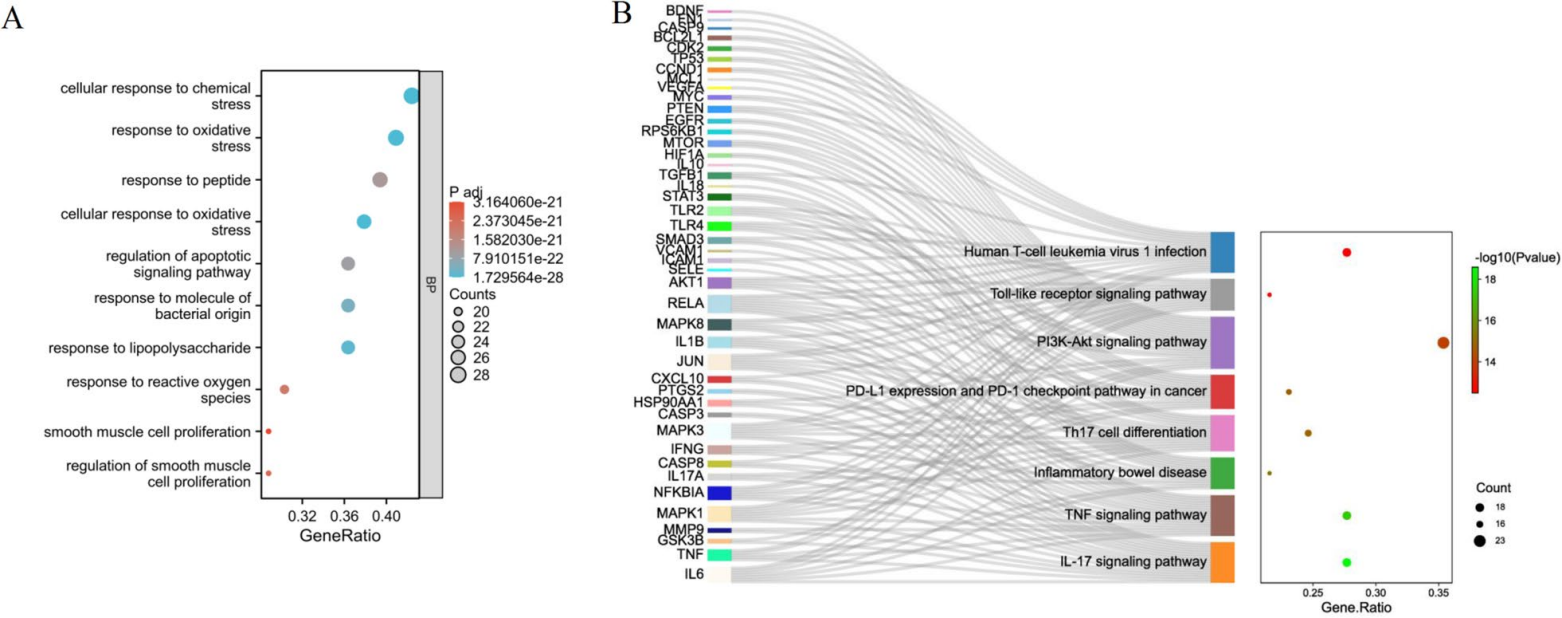
Enrichment analysis of cluster 1. **A** Bubble diagram depicted the top 10 GO-BP terms. **B** The Sankey diagram presented the relationship between potential targets and KEGG pathways (left). The bubble diagram depicted the top 8 KEGG pathways (right)

To further investigate the biological functions of putative targets of celastrol in the treatment of PSA, an enrichment analysis was performed. GO analysis results revealed that the genes in cluster 1 were significantly enriched in biological processes, including cellular response to chemical stress, response to oxidative stress, response to peptide, cellular response to oxidative stress, regulation of apoptotic signaling pathway, etc. (Fig. 7A). The KEGG analysis results indicated that these genes were mainly enriched in human T cell leukemia virus 1 infection, toll-like receptor signaling pathway, PI3K-AKT signaling pathway, PD-L1 expression and PD-1 checkpoint pathway in cancer, Th17 cell differentiation, inflammatory bowel disease, TNF signaling pathway, IL-17 signaling pathway, colorectal cancer, etc. (Fig. 7B).

### Molecular docking analysis

**Fig. 8:**
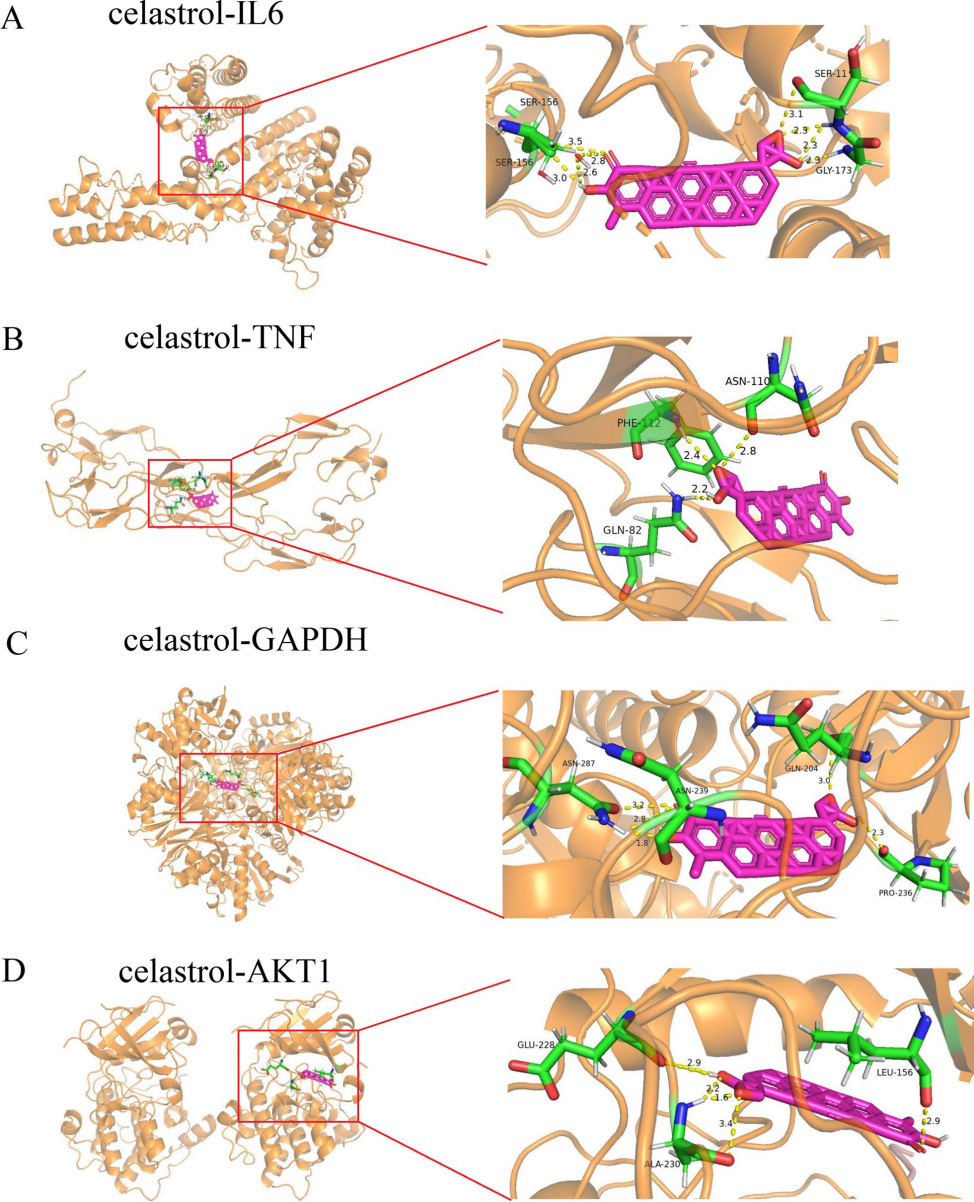
Molecular docking results of celastrol with core targets. **A** Celastrol-IL6; **B** Celastrol-TNF; **C** Celastrol-GAPDH; **D** Celastrol-AKT1

We performed molecular docking to analyze the binding of the core targets (IL6, TNF, GAPDH, and AKT1) and celastrol. Binding energies were all less than − 5.0 kcal/mol, and the results were shown in Table 1. In detail, celastrol bound to IL6 with a binding energy of − 15.76 kcal/mol, TNF at − 12.94 kcal/mol, GAPDH at − 13.77 kcal/mol, and AKT1 at − 11.9 kcal/mol. These results revealed that celastrol had a strong affinity for key target proteins and all have good binding stability. Figure 8 presented the molecular docking patterns of celastrol with core target proteins.

**Table 1:** The results of molecular docking

Drug name	Target	Binding energy
Celastrol	IL6	− 15.76
Celastrol	TNF	− 12.94
Celastrol	GAPDH	− 13.77
Celastrol	AKT1	− 11.9

### Celastrol alleviates inflammatory response in the mannan-induced PSA

**Fig. 9:**
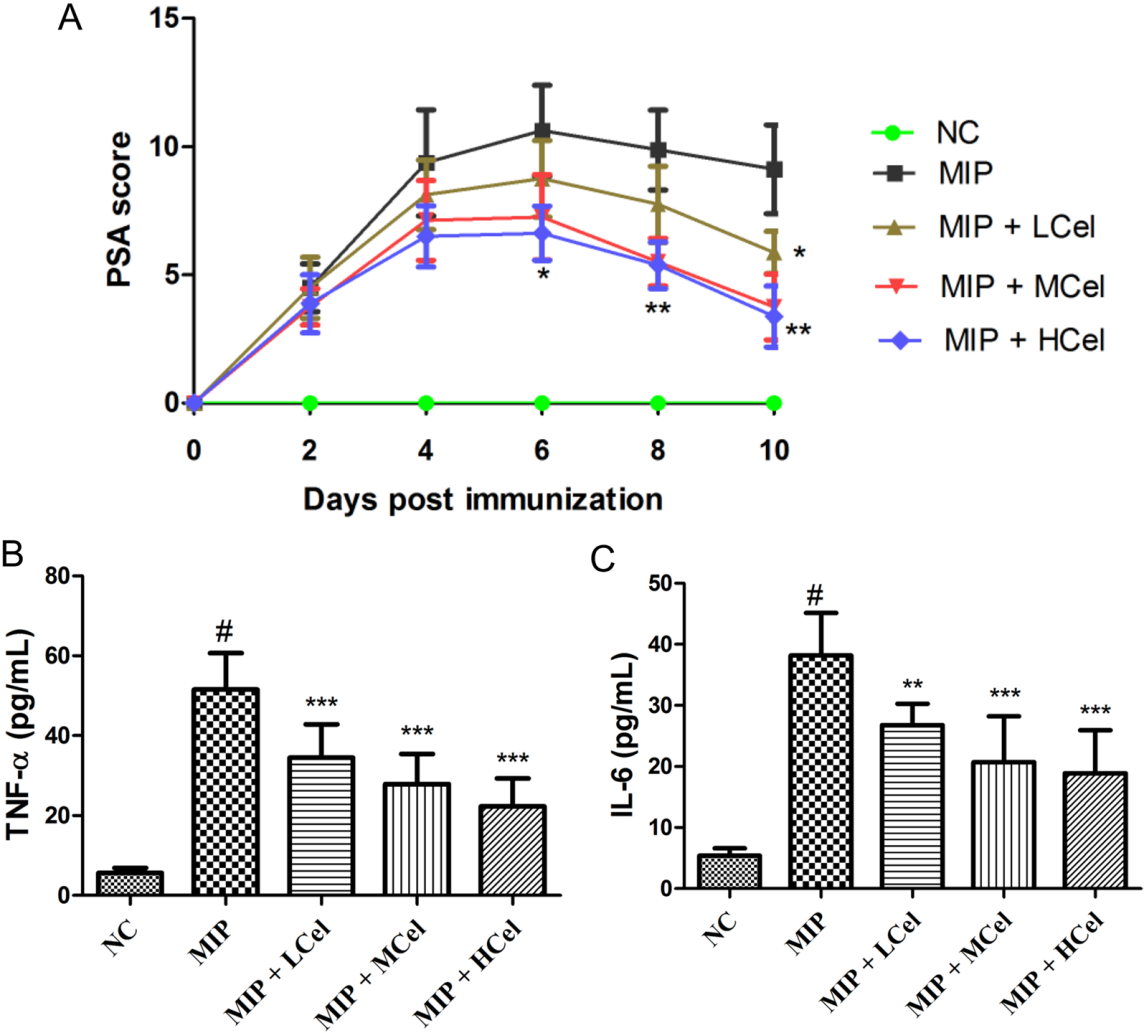
Celastrol alleviates inflammation in mannan-induced PSA mice. **A** Celastrol improved PSA symptoms in MIP group. The TNF-α (**B**) and IL-6 (**C**) levels in serum. #*p* < 0.001, the MIP group compared to the NC group; **p < 0.01, ***p < 0.001, the therapeutic group compared to the MIP group

**Fig. 10:**
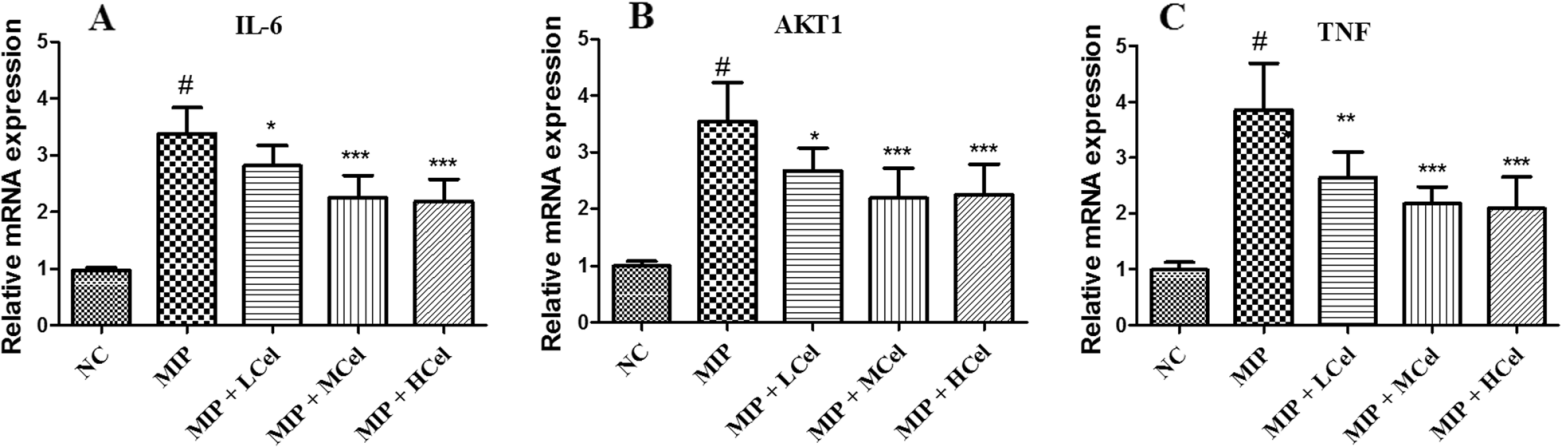
Verification of core genes expression in mannan-induced PSA mice. The expression of IL-6 (**A**), AKT1 (**B**), and TNF (**C**) genes was measured by qRT-PCR. #*p* < 0.001, the MIP group compared to the NC group; *p < 0.05, **p < 0.01, ***p < 0.001, the therapeutic group compared to the MIP group

As shown in Fig. 9A, after day 8, the group treated with celastrol (1 mg/kg and 2 mg/kg) showed a significant decrease in PSA score compared to the MIP group (*p* < 0.01). In addition, celastrol inhibited TNF-α (Fig. 9B) and IL-6 (Fig. 9C) levels in the mannan-induced PSA groups (*p* < 0.01). Furthermore, we also carried out qRT-PCR analysis to validate the results of bioinformatics analysis. As shown in Fig. 10, after treatment with celastrol (1 mg/kg and 2 mg/kg), the expressions of IL-6, AKT1, and TNF displayed a significant down-regulation in the MIP group (*p* < 0.01). Our study indicated that celastrol has the potential to reduce inflammation in mice that were stimulated with mannan. These results align with the findings from the bioinformatics analysis.

## Discussion

PSA is a common and recurrent chronic inflammatory arthropathy with a genetic predisposition, and the main pathogenesis involves the immune-mediated proliferation of keratinocytes [3]. The prevention and early treatment of PSA remain a challenge because the pathogenesis of the disease is still unclear [26, 27]. In recent years, microarray technology has been widely used for the prediction of potential diagnostic and therapeutic targets for various diseases and has the potential to be an effective tool for the early diagnosis and exploration of therapeutic targets for diseases [28–31]. In this study, 734 DEGs in PSA, including 497 up-regulated and 237 down-regulated genes, were obtained by bioinformatic analysis of the GSE61281 dataset in the GEO database. After performing WGCNA analysis, a PSA core gene, CLEC2B, was obtained. CLEC2B is a member of the superfamily encoding C-type lectin/C-type lectin-like structural domains. Intercellular signaling, glycoprotein conversion, and their role in inflammation are just some of the functions of these family members [32]. In addition, CLEC2B is a prognostic biomarker in cancer, including endometrial cancer and melanoma [33, 34]. In our study, CLEC2B was significantly increased in PSA. To further verify this, we collected blood samples from PSA patients for qRT-PCR and found that CLEC2B was highly expressed in PSA patients and was statistically significant (*p* < 0.01). It is therefore believed that CLEC2B is likely to be a biomarker for the diagnosis of PSA.

GSEA results indicated that DEGs were mainly involved in immune processes (leukocyte transendothelial migration, acute myeloid leukemia, T cell receptor signaling pathway, and B cell receptor signaling pathway). Previous studies indicated that synovial B cells play an important role in the pathogenesis of PSA [35, 36]. In addition, the immune cell infiltration results revealed that CD4 + memory T cells and aDC levels were significantly increased in PSA patients. T cells may play a central role in the maintenance and amplification of the pathogenic inflammatory cycle in psoriatic plaques, leading to the major clinical manifestations of PSA [37]. Increased levels of expression of DC from patients with PSA are involved in the immune response against intracellular bacteria [38]. These findings also revealed that PSA is an immune process involving multiple immune cells.

Another important finding of this study was that CMap analysis results identified celastrol as a candidate drug for PSA, and network pharmacology analysis suggested that celastrol may be able to treat PSA partly through the regulation of immune- and inflammation-related pathways, including Th17 cell differentiation, TNF signaling pathway, and IL-17 signaling pathway, etc. PSA is an inflammatory musculoskeletal disease that is related to cutaneous psoriasis [1]. IL-17 is the major effector cytokine in PSA pathogenesis [7, 39]. Disruption of the IL-23/Th17 axis induces Th17-mediated inflammation, resulting in skin and synovial inflammation and bone pathology highly reminiscent of human PSA [40]. In addition, IL6, TNF, GAPDH, and AKT1 were identified as the core target proteins using 11 algorithms in CytoHubba. IL6 is implicated in the pathogenesis of chronic inflammatory diseases and is a key cytokine in the acute phase response [41]. In patients with psoriasis, a high level of IL6 gene expression may be a marker for possible joint damage [42]. TNF-alpha and IL-6 are key pathogens involved in immune-mediated bone diseases, such as postmenopausal osteoporosis and rheumatoid arthritis [43]. TNF-alpha has been identified as a promising therapeutic target for the treatment of PSA [44]. GAPDH is a key enzyme for glycolysis, which plays a vital role in metabolism [45]. Akt1 is involved in survival migration, cell metabolism, and acute inflammation [46]. Celastrol plays an important role in treating various inflammation-related diseases, particularly in regulating immune or non-immune cell signaling in the inflammatory microenvironment [47]. Arthritis and another autoimmune disease may also benefit from celastrol therapy [48]. Our research has been greatly encouraged by these studies on the treatment of inflammatory diseases with celastrol. In our study, molecular docking was performed to validate the possible bindings between celastrol and core target proteins. Thus, we speculated that celastrol may regulate core target proteins (IL6, TNF, GAPDH, and AKT1) to alleviate the inflammatory response in PSA.

## Conclusion

In summary, in this study, CLEC2B was a diagnostic marker for PSA patients. In addition, the CMap database identifies celastrol as a potential drug for the treatment of PSA through the inhibition of inflammatory responses. However, whether celastrol can be a natural drug for PSA and the exact mechanism of its action still needs to be investigated and proven experimentally, and our results will hopefully provide new clinical insights.

## Supplementary Information

**Additional file 1: Table S1.** Sequences of primers used in the present study. **Table S2.** Hub genes identified by WGCNA. **Table S3.** Screening for potential anti-PSA natural active ingredients by CMap. **Fig. S1.** Identification of core targets. 11 algorithms in CytoHubba were used to identify core targets.

## Data Availability

All data used in the present study were available from the corresponding author upon reasonable request.
