# Supplementary material for: Discovery of CLEC2B as a diagnostic biomarker and screening of celastrol as a candidate drug for psoriatic arthritis through bioinformatics analysis
Source: J Orthop Surg Res. 2023 May 29;18:390. doi: 10.1186/s13018-023-03843-0 (PMC10226212; doi:10.1186/s13018-023-03843-0)
Supplement: Supplementary file 1 — Additional file 1: Table S1. Sequences of primers used in the present study. Table S2. Hub genes identified by WGCNA. Table S3. Screening for potential anti-PSA natural active ingredients by CMap. Fig. S1. Identification of core targets. 11 algorithms in CytoHubba were used to identify core targets. [file 13018_2023_3843_MOESM1_ESM.docx]

Supplementary tables

Table S1 Sequences of primers used in the present study.

| Gene | Forward primer (5' to 3') | Reverse primer (5' to 3') |
| --- | --- | --- |
| CLEC2B | GTTCCACTCAACATGCCGAC | TGCCATCTTCAGTCCAATCCA |
| IL-6 | TCCTACCCCAACTTCCAATGCTC | TTGGATGGTCTTGGTCCTTAGCC |
| TNF | CTGGAAATGYCTSAGCATCTGT | GACWGCWCCTTGGTAGATGGA |
| AKT1 | TGGACTACTTGCACTCCGAGAA | TTATCTTGATATGCCCGTCCTT |
| β-ACTIN | ACCACAGTCCATGCCATCAC | TCCACCACCCTGTTGCTGTA |

Table S2 Hub genes identified by WGCNA.

| Gene | Module | MM_R | MM_pvalue | GS_R | GS_pvalue |
| --- | --- | --- | --- | --- | --- |
| ACTR6 | blue | 0.950468 | 9.03E-17 | 0.607123 | 0.000229 |
| CIAO2A | blue | 0.840128 | 1.82E-09 | 0.612833 | 0.000192 |
| CKLF | blue | 0.835203 | 2.77E-09 | 0.604633 | 0.000247 |
| CLEC2B | blue | 0.871324 | 8.71E-11 | 0.639613 | 8.10E-05 |
| COX16 | blue | 0.848893 | 8.29E-10 | 0.646945 | 6.30E-05 |
| CSTA | blue | 0.884186 | 1.96E-11 | 0.609381 | 0.000214 |
| DPM1 | blue | 0.89334 | 6.08E-12 | 0.606535 | 0.000233 |
| GEMIN2 | blue | 0.909301 | 5.96E-13 | 0.606254 | 0.000235 |
| GNL3 | blue | 0.856668 | 3.97E-10 | 0.676398 | 2.14E-05 |
| HAT1 | blue | 0.833823 | 3.10E-09 | 0.628441 | 0.000117 |
| HAUS1 | blue | 0.808675 | 2.15E-08 | 0.608891 | 0.000217 |
| HNMT | blue | 0.807943 | 2.26E-08 | 0.696631 | 9.48E-06 |
| MRPL13 | blue | 0.821494 | 8.32E-09 | 0.622922 | 0.00014 |
| MRPL47 | blue | 0.813874 | 1.47E-08 | 0.644583 | 6.84E-05 |
| MRPS18C | blue | 0.823454 | 7.15E-09 | 0.61787 | 0.000165 |
| MRPS18CP3 | blue | 0.802402 | 3.33E-08 | 0.60207 | 0.000267 |
| PDCD10 | blue | 0.886604 | 1.45E-11 | 0.615225 | 0.000179 |
| POLR2K | blue | 0.83332 | 3.24E-09 | 0.640756 | 7.79E-05 |
| PSMC6 | blue | 0.921627 | 7.25E-14 | 0.623549 | 0.000137 |
| RPL23 | blue | 0.803019 | 3.19E-08 | 0.637269 | 8.77E-05 |
| RPL9 | blue | 0.841082 | 1.67E-09 | 0.695027 | 1.01E-05 |
| RSL24D1 | blue | 0.906186 | 9.69E-13 | 0.644791 | 6.79E-05 |
| RWDD1 | blue | 0.829093 | 4.57E-09 | 0.6255 | 0.000129 |
| TAF9 | blue | 0.857609 | 3.62E-10 | 0.644164 | 6.94E-05 |
| TMEM126B | blue | 0.932274 | 8.72E-15 | 0.626743 | 0.000124 |
| TRIAP1 | blue | 0.835468 | 2.70E-09 | 0.616526 | 0.000172 |
| TRMT11 | blue | 0.917311 | 1.57E-13 | 0.606054 | 0.000237 |
| ZFAND1 | blue | 0.940243 | 1.41E-15 | 0.625714 | 0.000128 |

Table S3 Screening for potential anti-PSA natural active ingredients by CMap.

| Rank | Score | Name | Description |
| --- | --- | --- | --- |
| 1 | -99.58 | celastrol | Anti-inflammatory |
| 2 | -99.46 | prunetin | Breast cancer resistance protein inhibitor |
| 3 | -98.13 | piceid | Glucosidase inhibitor |
| 4 | -96.85 | securinine | GABA receptor antagonist |
| 5 | -96.19 | strychnine | Acetylcholine receptor antagonist |
| 6 | -95.1 | arecaidine | Acetylcholine receptor agonist |
| 7 | -94.33 | farnesol | FXR agonist |
| 8 | -92.57 | solanine | Acetylcholinesterase inhibitor |
| 9 | -91.2 | taurodeoxycholic-acid | Bile acid |

Supplementary figures


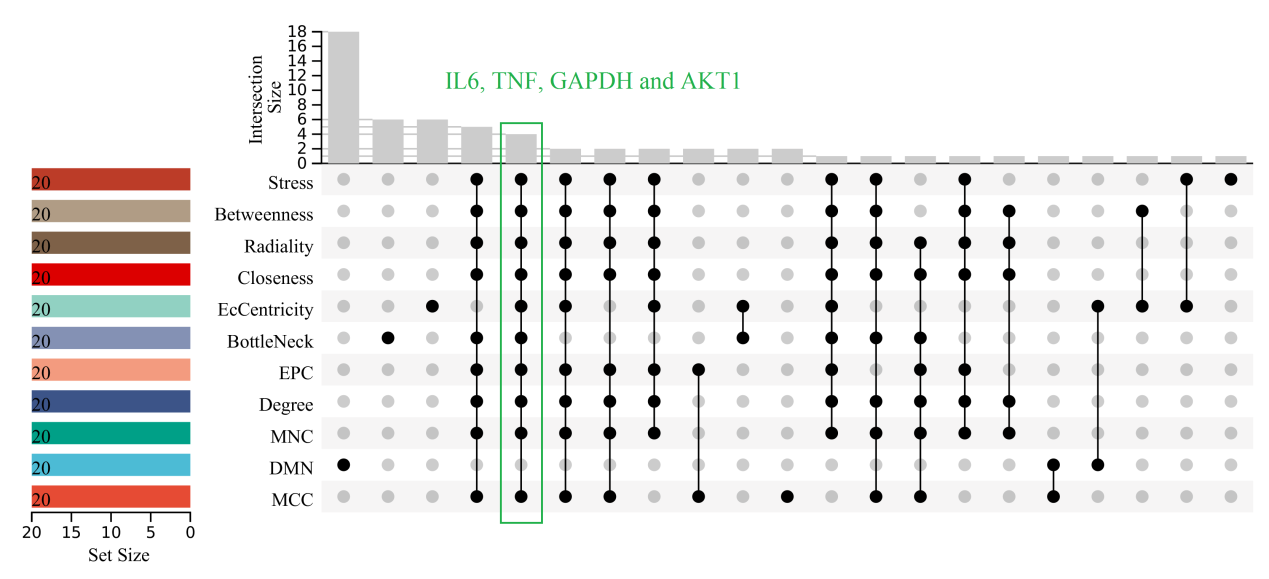


Figure S1 Identification of core targets. 11 algorithms in CytoHubba were used to identify core targets.
